# Exploring 2D and 3D radiomic models for predicting microvascular invasion in hepatocellular carcinoma: a novel perspective on tumor heterogeneity

**DOI:** 10.3389/fonc.2025.1590655

**Published:** 2025-09-17

**Authors:** Zexin Yin, Meilong Wu, Youyao Li, Zhike Li, Shiyun Bao, Liping Liu

**Affiliations:** ^1^ Department of Pancreatic Center, Guangdong Provincial Hospital of Chinese Medicine, Guangzhou, Guangdong, China; ^2^ Department of Hepatobiliary and Pancreatic Surgery, The Second Clinical Medical College of Jinan University, Shenzhen People’s Hospital, Shenzhen, Guangdong, China

**Keywords:** microvascular invasion, intratumoral heterogeneity, radiomics, two-dimensional and three-dimensional models, machine learning

## Abstract

**Objective:**

This study aims to develop models for predicting microvascular invasion (MVI) in hepatocellular carcinoma (HCC) patients prior to surgery using two-dimensional (2D) and three-dimensional (3D) radiomics features from contrast-enhanced computed tomography (CT). The study compares the predictive performance of various models and explores the potential of radiomics to capture tumor spatial heterogeneity.

**Materials and methods:**

A total of 150 hepatocellular carcinoma (HCC) patients who underwent contrast-enhanced CT examination and curative resection were included in this study. 2D features from the largest cross-sectional slice, as well as 3D radiomic features, were extracted from the non-contrast (NC), arterial phase (AP), portal venous phase (PVP), and balanced phase (BP) images. Feature selection was performed using the least absolute shrinkage and selection operator (LASSO) algorithm, and predictive models were constructed using logistic regression and XGBoost machine learning algorithms. The predictive performance of the models was evaluated using the area under the receiver operating characteristic curve (AUC).

**Results:**

The 2D BP model (AUC = 0.801) and 3D PVP model (AUC = 0.876) showed superior performance among single-sequence models. The 2D multi-sequence model (AUC = 0.851) outperformed the 3D combined model (AUC = 0.811). Radiomics-based models outperformed clinical feature-based models, and combining radiomics scores with clinical features improved prediction accuracy. However, 3D models did not significantly outperform 2D models.

**Conclusion:**

Both 2D and 3D radiomics models are effective for predicting MVI in HCC patients preoperatively. While the 3D model captures spatial heterogeneity, the 2D model excels at capturing local texture features. This study provides new insights into radiomics in HCC, contributing to its clinical application and standardization.

## Introduction

Hepatocellular carcinoma (HCC) ranks as the sixth most common malignancy and the third leading cause of cancer-related mortality worldwide (1). The World Health Organization predicts that there will be over 1 million new liver cancer cases by 2025 (2). Surgical resection remains the most effective treatment for early-stage HCC; however, postoperative recurrence occurs in up to 70% of patients, significantly impacting long-term survival (3). Predicting HCC recurrence preoperatively and implementing targeted interventions are pressing and clinically significant.

Plenty of studies have demonstrated that microvascular invasion (MVI) is an independent risk factor for postoperative recurrence of HCC (4). As a highly vascularized tumor characterized by dual blood supply, HCC is prone to MVI, which represents the most critical pathological mechanism underlying postoperative recurrence and metastasis. MVI is also one of the key factors in predicting HCC recurrence (5). Furthermore, MVI is associated with patient prognosis and serves as an independent risk factor for both postoperative survival and extrahepatic metastasis in HCC patients (6).

In recent years, with the deepening of research on MVI and advances in radiomics, the precise preoperative prediction of MVI has become feasible. Radiomics, an emerging field, was first introduced in 2012 by Dutch scholar Lambin et al. (7). Over the past decade, radiomics has rapidly developed to be a bridge between medical imaging and precision medicine.

Enhanced computed tomography (CT) is one of the most commonly employed diagnostic tools for preoperative evaluation in HCC patients. Prediction models based on radiomic features derived from enhanced CT images have been widely recognized for their efficiency in preoperatively predicting MVI in HCC (8, 9). However, there are discrepancies in specific methodologies and detailed approaches in existing studies.

In radiomics-based region of interest (ROI) delineation, two main methods are used: contouring all tumor-involved slices [three-dimensional(3D)] and contouring only the single axial slice with the largest tumor area [two-dimensional(2D)]. The advantage of 3D ROI is evident, as it enables comprehensive analysis of all tumor cell populations, including spatial distribution within the tumor, which has been the predominant method in most previous studies. In contrast, 2D ROI provides clearer tumor boundaries and greater reproducibility (10). Moreover, 2D ROI is easier to obtain and requires significantly less workload and computational resources compared to 3D ROI. Given the extensive spatial heterogeneity observed within HCC tumors (11), it remains unclear whether single-layer images representing the largest tumor cross-sectional area can adequately reflect the overall tumor characteristics in radiomics studies. To date, no studies in the field of HCC have reported performance differences between 2D and 3D feature-based models, and further investigations are warranted.

The aim of this study was to develop radiomics-based predictive models using 2D and 3D features derived from different phases of enhanced CT imaging to preoperatively predict MVI in HCC patients. Additionally, the study sought to compare the predictive performance of these models and explore whether radiomics can capture tumor spatial heterogeneity. This work aimed to standardize the workflow of radiomics research in HCC and address the methodological inconsistencies observed in prior radiomics studies.

## Materials and methods

### Patient selection

This retrospective study utilized clinical information and CT images obtained from the electronic medical record system and imaging data system of Shenzhen People’s Hospital. The study was approved by the Institutional Review Board of Shenzhen People’s Hospital, with a waiver of informed consent. Consecutive patients who underwent contrast-enhanced CT scans at our hospital between July 2015 and June 2022 were included. The sample size was determined based on feasibility. All patients who met the eligibility criteria were retrospectively collected. The inclusion criteria required that all imaging data be uniformly reformatted using a B-spline interpolation resampling algorithm (12)with a voxel size of 1.0 mm × 1.0 mm × 1.0 mm. The exclusion criteria were as follows: (1) patients receiving preoperative antitumor treatments; (2) patients with a history of recurrent HCC or concurrent primary tumors; (3) patients with distant metastases associated with HCC; (4) poor image quality; and (5) incomplete clinical or pathological information.

“Poor image quality” was defined as the presence of significant motion artifacts, low signal-to-noise ratio, incomplete coverage of the region of interest (ROI), or other technical issues that hindered accurate segmentation and feature extraction. Image quality was independently assessed by two experienced radiologists, and any discrepancies were resolved via consensus.

After screening, a total of 150 HCC patients were included in the study (120 males and 30 females, mean age: 57 years, range: 29–84 years), comprising 46 MVI-positive cases and 104 MVI-negative cases. Clinical characteristics, including gender, age, carcinoembryonic antigen (CEA), alpha fetoprotein (AFP), CA125, and 44 other variables, were extracted from the electronic medical record system. Patients were randomized to the training and validation cohorts in a 70:30 ratio. Demographic and clinical characteristics of all patients are summarized in **Table 1**.

**Table 1:** Baseline characteristics of patients (grouped by clinical relevance).

Variables	Total (n=150)	Training cohort(n=106)	Testing cohort (n=44)	p-value
Demographics				
▪ Gender, n (%)				0.226
Male	30 (20)	18 (17)	12 (27)	
Female	120 (80)	88 (83)	32 (73)	
▪ Age, Mean ± SD	56.99 ± 11.72	57.53 ± 11.39	55.7 ± 12.53	0.407
▪ BMI, Median (IQR)	23.27 (21.02-25.62)	23.2 (21.01-25.89)	23.55 (21.66-25.07)	0.828
Liver disease status				
▪ Liver Cirrhosis, n (%)				1.000
No	72 (48)	51 (48)	21 (48)	
Yes	78 (52)	55 (52)	23 (52)	
▪ HBsAg, n (%)				0.687
Negative	43 (29)	32 (30)	11 (25)	
Positive	106 (71)	73 (69)	33 (75)	
Not tested	1 (1)	1 (1)	0 (0)	
▪ HCVAb, n (%)				0.631
Negative	145 (97)	103 (97)	42 (95)	
Positive	5 (3)	3 (3)	2 (5)	
Tumor characteristics				
▪ MVI, n (%)				1.000
Absent	104 (69)	73 (69)	31 (70)	
Present	46 (31)	33 (31)	13 (30)	
▪ Tumor Size, mm, Median (IQR)	40.5 (25-62)	35.5 (24-61)	50.5 (28-66.25)	0.088
Hematological parameters				
▪ Hemoglobin, Mean ± SD	140.31 ± 17.39	141.2 ± 18.59	138.18 ± 14.06	0.281
▪ Platelets, Median (IQR)	192 (138-233.25)	200.5 (152.25-242.75)	171.5 (126-197)	0.004
▪ Lymphocytes, Median (IQR)	1.67 (1.27-2.2)	1.69 (1.32-2.28)	1.66 (1.17-2.02)	0.638
▪ Neutrophils, Median (IQR)	3.15 (2.4-4.19)	3.32 (2.46-4.5)	2.82 (2.3-3.55)	0.030
▪ Monocytes, Median (IQR)	0.48 (0.37-0.64)	0.5 (0.38-0.64)	0.44 (0.32-0.62)	0.139
Liver function tests				
▪ Albumin, Median (IQR)	41.75 (39.12-44.4)	42.25 (39.7-44.65)	40.65 (38.42-43.23)	0.077
▪ Total Protein, Median (IQR)	69.85 (66.82-73.4)	69.95 (67.25-73.88)	69.75 (66.75-72.03)	0.356
▪ Total Bilirubin, Median (IQR)	12.65 (9.12-16.48)	13.7 (9.5-16.48)	11.95 (8.9-15.98)	0.521
▪ Direct Bilirubin, Median (IQR)	4.56 (3.34-5.9)	4.94 (3.35-5.9)	4.06 (3.34-5.78)	0.234
▪ Indirect Bilirubin, Median (IQR)	7.96 (5.56-11.04)	7.88 (5.77-11.09)	8.1 (5.15-10.53)	0.740
▪ ALT, Median (IQR)	30 (23-40.75)	29 (23-40.75)	31.5 (22.75-40.25)	0.812
▪ AST, Median (IQR)	29 (23-39.5)	28.5 (23.25-37.75)	30 (22-41.75)	0.841
▪ GGT, Median (IQR)	36.5 (26-72.5)	36 (25-63.5)	37.5 (26.75-98)	0.300
▪ ALP, Median (IQR)	74 (61-94)	73 (61-94)	76 (63-96.25)	0.857
▪ LRF_15min, Median (IQR)	4.3 (2.82-6.38)	4.25 (2.5-6.7)	4.45 (2.9-6.1)	0.740
Coagulation profile				
▪ PT, Median (IQR)	12.15 (11.53-13.07)	12.15 (11.5-13.07)	12.15 (11.67-12.95)	0.833
▪ APTT, Median (IQR)	31.95 (29.63-34.5)	31.8 (29.6-34.48)	32.15 (30.08-34.5)	0.825
▪ TT, Median (IQR)	16.2 (15-17.38)	16 (14.9-17.28)	16.5 (15.7-17.45)	0.072
▪ INR, Median (IQR)	1 (0.96-1.06)	0.99 (0.95-1.06)	1.02 (0.98-1.06)	0.126
▪ Fibrinogen, Median (IQR)	2.92 (2.53-3.51)	2.88 (2.48-3.54)	3.04 (2.64-3.43)	0.537
Metabolic parameters				
▪ Blood Glucose, Median (IQR)	5.08 (4.89-5.64)	5.08 (4.91-5.73)	5.08 (4.82-5.33)	0.358
▪ BUN, Median (IQR)	4.94 (4.08-5.85)	5.06 (4.11-5.96)	4.39 (3.7-5.52)	0.056
▪ Creatinine, Median (IQR)	27 (24-30.23)	27 (24-30)	28.5 (24-32)	0.624
Tumor markers				
▪ AFP, Median (IQR)	29.15 (3.48-297.15)	29.15 (3.4-316.8)	28.85 (3.59-267.85)	0.785
▪ CEA, Median (IQR)	2.02 (1.38-2.92)	2.02 (1.31-2.95)	2.12 (1.51-2.68)	0.964
▪ CA125, Median (IQR)	10.38 (6.56-15.48)	10.13 (6.56-14.83)	11.16 (7.07-17.45)	0.355
▪ CA199, Median (IQR)	16.74 (9.63-28.91)	17.06 (9.54-29.09)	16.3 (11.52-26.62)	0.964
Composite indices				
▪ Lymphocyte/Monocyte Ratio, Mean ± SD	3.7 ± 1.44	3.64 ± 1.47	3.83 ± 1.35	0.457
▪ NLR, Median (IQR)	1.89 (1.35-2.57)	1.98 (1.43-2.66)	1.7 (1.3-2.52)	0.326
▪ PLR, Median (IQR)	109.46 (81.82-141.52)	113.5 (87.28-157.66)	92.49 (74.84-123.62)	0.031
▪ SII, Median (IQR)	356.97 (192.94-552.94)	393.5 (252.71-611.15)	287.65 (168.45-424.92)	0.010
▪ APRI, Median (IQR)	0.36 (0.25-0.58)	0.34 (0.24-0.53)	0.42 (0.28-0.73)	0.096
▪ PNI, Median (IQR)	51.17 (46.71-53.33)	51.65 (47.24-53.74)	49.95 (46.05-52.55)	0.121
▪ ALRI, Median (IQR)	17.93 (11.47-27.47)	16.95 (11.29-27.16)	19.04 (12.49-30.43)	0.450
▪ AST/Neutrophil Ratio, Median (IQR)	9.59 (6.38-15.19)	9.38 (6.15-15.02)	10.41 (6.68-15.67)	0.279
▪ LF Index, Median (IQR)	1.67 (1.07-2.78)	1.6 (1.05-2.63)	2.23 (1.24-2.95)	0.090

Group comparisons for continuous and categorical variables were performed using TableOne, with the automatic selection of appropriate descriptive methods (e.g., interquartile range, frequency, and percentage, mean and standard deviation) and statistical tests (such as t-test, Mann-Whitney U test, Chi-square test, or Fisher’s exact test).

### CT imaging and ROI segmentation

All CT examinations were performed using spiral CT scanners from the Radiology Department of Shenzhen People’s Hospital, with identical scanning parameters. The imaging protocol adhered to the standards recommended by the LI-RADS guidelines (13). 3D manual segmentation was conducted using 3D Slicer software by a hepatobiliary surgeon with nine years of clinical experience. ROIs were delineated along the visible boundaries of the lesions in all non-contrast (NC), arterial phase (AP), portal venous phase (PVP), and balanced phase (BP) images, encompassing the entire lesion volume. Incorporating peritumoral regions into the ROIs has been demonstrated in multiple studies to enhance the predictive performance of radiomics models (8, 14). Accordingly, a 3-mm margin was automatically expanded using 3D Slicer, with manual removal of any regions extending beyond the liver volume to ensure the final ROI encompassed the peritumoral area. The final segmentation results were validated by a senior hepatobiliary surgeon with 14 years of clinical experience. Subsequently, the software automatically generated 2D images of the tumor’s largest cross-sectional area. To assess intra-observer reproducibility, a subset of ROIs was randomly re-annotated by the same hepatobiliary surgeon on the imaging dataset. Intraclass correlation coefficient (ICC) analysis demonstrated good consistency between the two sets of annotations.

### Radiomics feature extraction

Radiomics features were extracted using the Radiomics plugin package in 3D Slicer 5.4.0 for each phase: NC, AP, PVP, and BP. The B-spline interpolation resampling algorithm (12) was applied to standardize the image format, with a voxel spacing of 1.0 mm × 1.0 mm × 1.0 mm. The same extraction procedure was applied to wavelet-transformed derivative images (15) to enhance the dimensionality of the image data.

Each dataset comprised 1,130 features, with a one-to-one correspondence established to ensure comparability. These features included first-order shape features, first-order statistical features, second-order texture features, higher-order features, and wavelet-transformed features, quantitatively representing the imaging information of the corresponding tumor regions. Prior to further processing, the extracted features were dimensionless. Before further processing, all extracted features were standardized by non-dimensionalization. The Min-Max scaler function in Python was applied to normalize the training dataset, and the same scaling parameters were subsequently applied to the validation set. This procedure was employed to accelerate model training and to mitigate the potential impact of disproportionately scaled features on model performance.

### Radiomics feature selection: embedded least absolute shrinkage and selection operator

Most of the extracted radiomic features were not associated with the outcome. LASSO was employed to select a small subset of features most relevant to the outcome from the vast number of extracted features. Additionally, among the outcome-associated features, multiple highly correlated features might exist. By introducing L1 regularization, LASSO reduces the regression coefficients of some features to zero, thereby automatically selecting key features and effectively preventing overfitting, addressing the issues arising from multicollinearity (16).

2D and 3D features from NC, AP, PVP, and BP images in the eight radiomic groups were selected via the LASSO algorithm. The most predictive features were selected for model construction. In addition, we combined all four imaging phases and separately identified the optimal features from the hybrid ROIs across 2D and 3D datasets. These selected features were then applied to the validation cohort to confirm model generalizability.

### Model construction and evaluation

Eight predictive models were initially constructed using the 2D and 3D features selected from NC, AP, PVP, and BP. Both traditional logistic regression and XGBoost machine learning algorithms were used. To optimize the hyperparameters of the XGBoost model, a grid search strategy was employed. The parameter set yielding the highest mean AUC on the validation cohort was selected for the final model. The model’s predictive performance for MVI was evaluated using the receiver operating characteristic (ROC) curve and the area under the curve (AUC). Subsequently, a combined radiomics model was constructed, which incorporated the 2D and 3D features from all four phases.

**Figure 1:**
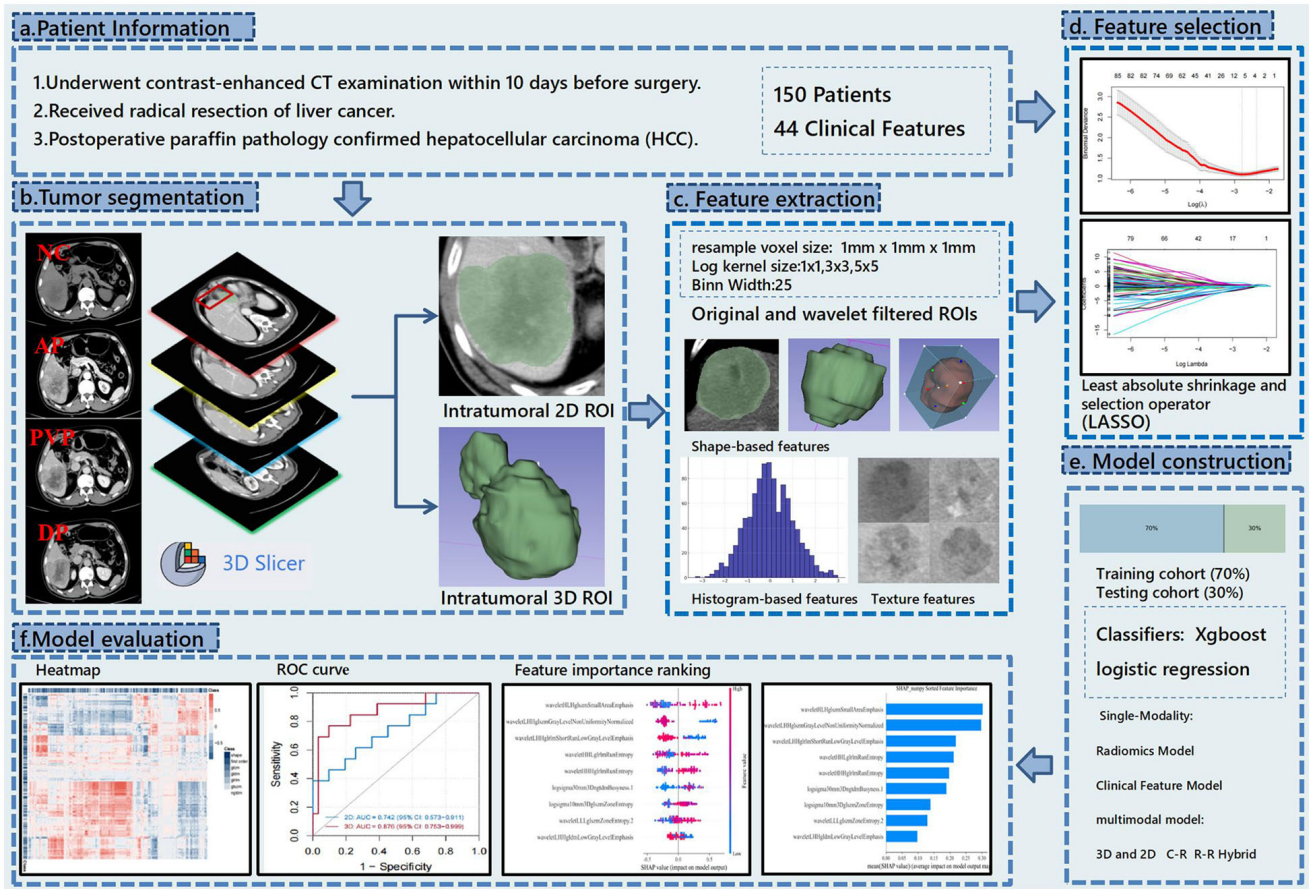
This flowchart illustrates the research process of predicting hepatocellular carcinoma (HCC) using CT radiomics. The study first collected preoperative contrast-enhanced CT data from 150 patients and performed tumor segmentation using 3D Slicer to label 2D and 3D regions. Then, shape, histogram, and texture features were extracted from both original and wavelet-filtered images. The most predictive features were selected using the LASSO method, and models were constructed using XGBoost and logistic regression with both single-modality (radiomics and clinical features) and multimodal models (combining 3D and 2D data). Finally, the model’s predictive performance was evaluated using ROC curves, heatmaps, and feature importance rankings.

Afterward, the optimal 2D and 3D radiomics-based models were selected from all the models. Radiomics scores were constructed using LASSO regression coefficients, and a combined model based on radiomics clinical features was further developed by combining the patient’s demographic and laboratory data, aiming to explore the best preoperative prediction approach for MVI. After the training phase, the model’s performance was evaluated in the test cohort. Shapley values (17) were then utilized to rank features and determine their contribution and importance. These results provided evidence for evaluating the intratumoral heterogeneity of HCC (**Figure 1**).

In this study, all statistical analyses were performed using the R language (version 4.4.1). Data preprocessing and descriptive statistics were conducted using the tidyverse and CBCgrps packages. Feature selection was performed through LASSO regression via the glmnet package, while cross-validation was performed using the caret package. The prediction models were constructed using xgboost, while hyperparameter tuning and model evaluation were carried out through caret. Model performance was assessed via AUC, and internal validation and calibration analysis were performed using the rms package. Model interpretation was facilitated through SHAP values, while the models were visualized using pheatmap and ggplot2. The 10-fold cross-validation was used for all analyses to ensure the robustness and generalizability of the models.

## Results

### Patient characteristics

A total of 150 eligible patients were included in this study. **Table 1** presents the statistical data and clinical characteristics of the training cohort (n = 106) and the testing cohort (n = 44). Histopathological examination revealed that the MVI status was balanced across the training and testing cohorts (p = 1).

### Extracted radiomic features

**Figure 2:**
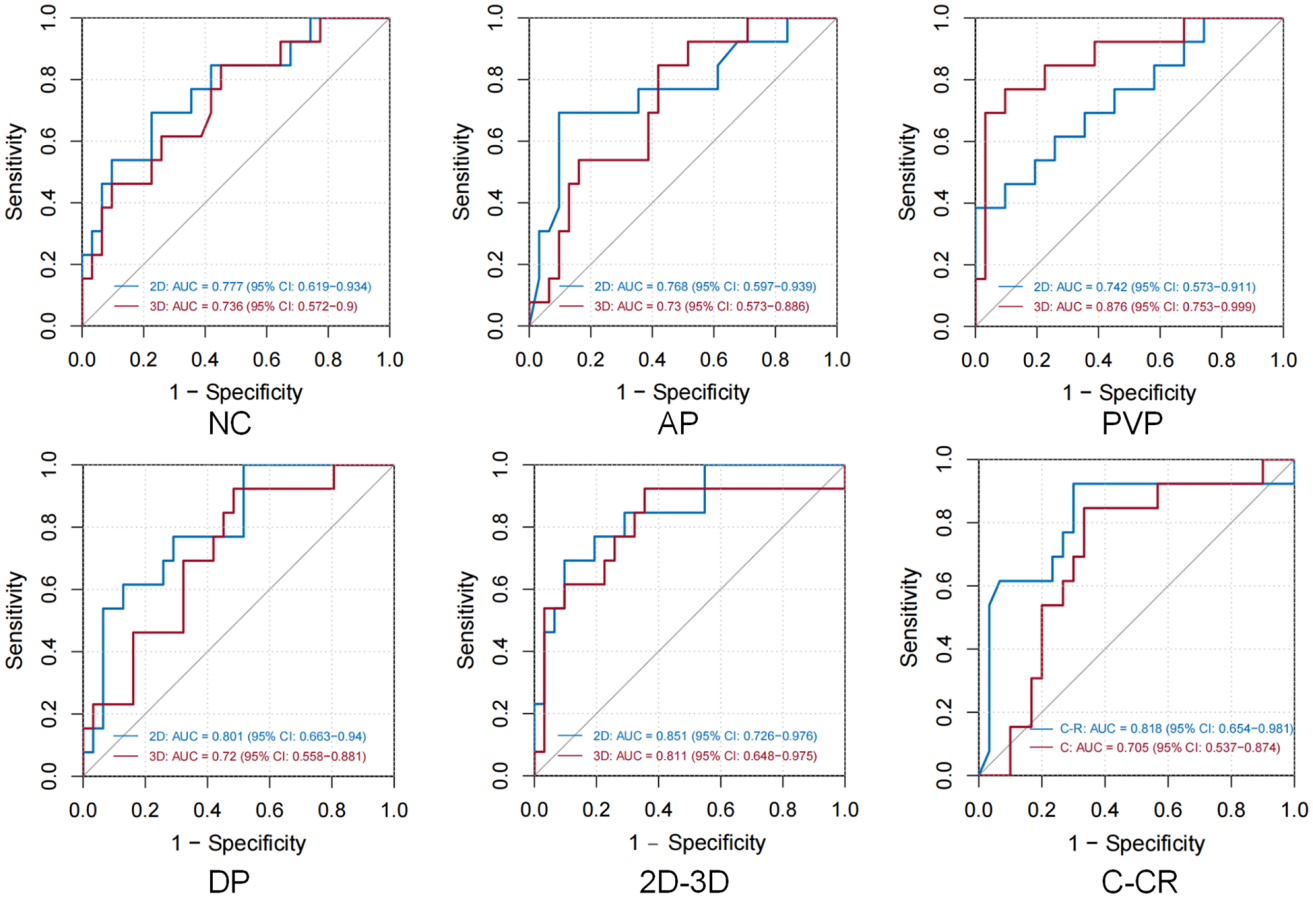
Illustrates the correlation of features in NC, AP, PVP, and BP in both 2D and 3D imaging modalities, with red indicating strong correlations and blue representing the inverse relationship. The features analyzed include Shape, First Order, GLCM (Gray Level Co-occurrence Matrix), GLDM (Gray Level Dependence Matrix), GLRLM (Gray Level Run Length Matrix), GLSZM (Gray Level Size Zone Matrix), and NGTDM (Neighboring Gray Tone Difference Matrix).

A total of 1130 features were extracted from eight sets of ROIs across four sequences in both 2D and 3D configurations. An unsupervised clustering algorithm was employed to explore the potential correlations among these features. Heatmaps were subsequently generated to visualize the correlations between 2D and 3D regions, as shown in **Figure 2**.

Our results indicated that the overall correlation between 2D and 3D features was weak; however, a small number of features from all four sequences demonstrated strong correlations. The correlation was strongest during the balance phase of 2D and 3D features, whereas the correlation during the PVP was weakest. These findings provided further insight into the interpretation of subsequent prediction model outcomes.

### Feature dimensionality reduction and selection

In the 2D ROI, seven, nine, six, and twelve MVI-related features were identified for the NC, AP, PVP, and BP, respectively. For the 3D ROI, the MVI-related features identified across these four phases were ten, six, six, and seven. Among the 63 features selected from the eight sets of imaging data, 41 were wavelet features.

### Performance and evaluation of predictive models

**Figure 3:**
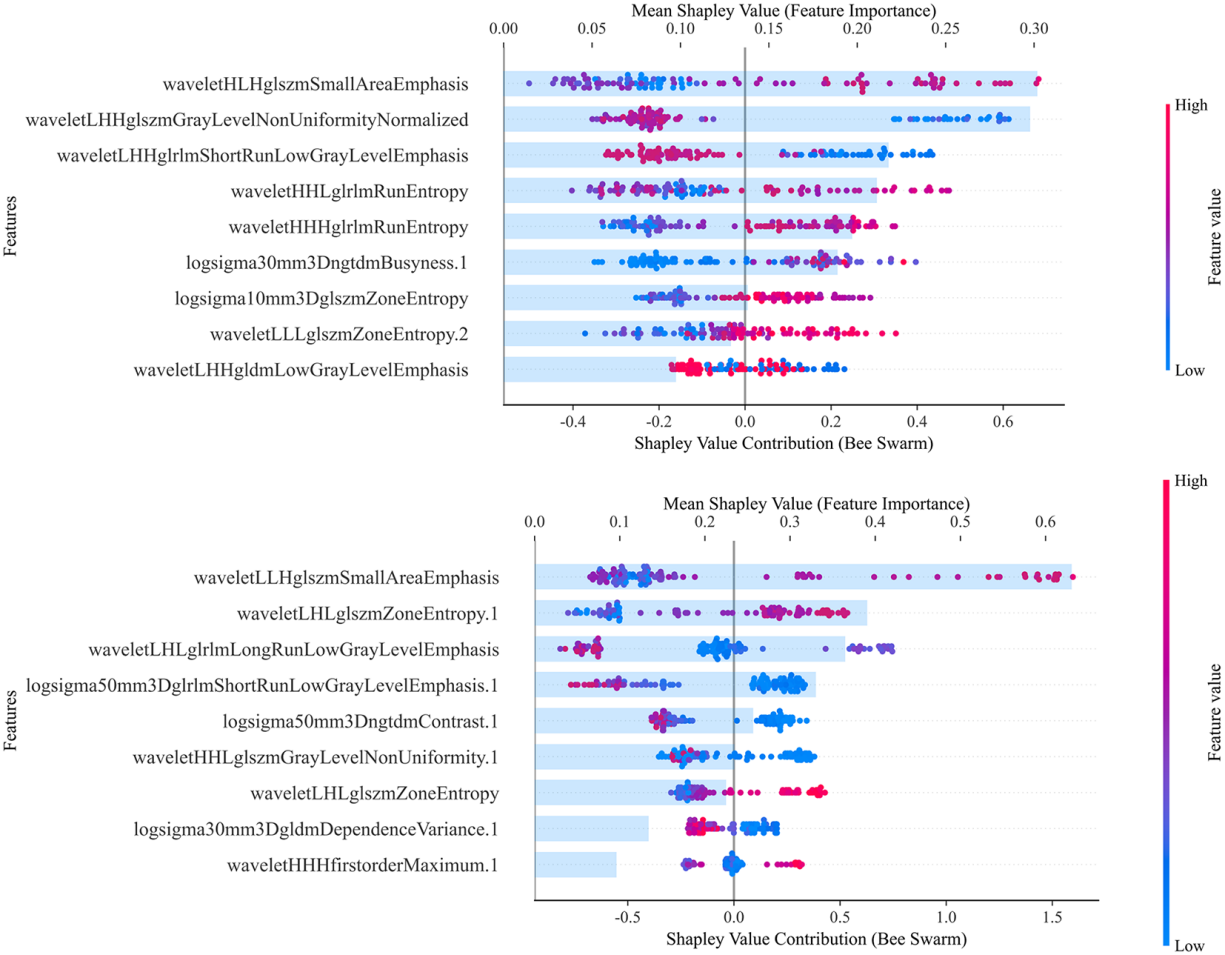
In all 12 groups of 24 logistic regression and XGBoost model test sets, the optimal modeling method was selected. Figures NC/AP/PVP/BP demonstrate the discriminative ability of different single-sequence 2D and 3D models, while the 2D-3D figure presents the ROC curves of the 2D and 3D combined radiomics models. The final figure shows the ROC curves for the C: Clinical Indicator Model and C-R: Clinical Indicator and Radiomics Combined Model.

Both logistic regression and machine learning methods were applied to all models. The performance of all 24 predictive models is summarized in **Table 2**, and the ROC curves of these models are depicted in **Figure 3**. In the models utilizing features extracted from single-sequence images in 16 cases, the 2D single-sequence model achieved the best AUC in the test set at the BP (logistic regression, AUC = 0.801, 95% CI: 0.663−0.94). The 3D single-sequence model exhibited the best AUC for the PVP (logistic regression, AUC = 0.876, 95% CI: 0.753−0.999).

Table 2Presents the predictive performance of all the models constructed in this study.

**Table:** 

(a)				
	2D train cohort	2D test cohort	3D train cohort	3Dtest cohort
Logistic regression				
NC	0.813 (0.733-0.892)	0.777 (0.619-0.934)	0.8 (0.715-0.885)	0.721 (0.561-0.881)
AP	0.789 (0.694-0.883)	0.736 (0.565-0.913)	0.825 (0.74-0.91)	0.73 (0.573-0.886)
PVP	0.762 (0.671-0.853)	0.739 (0.545-0.934)	0.789 (0.715-0.881)	0.876 (0.753-0.999)
BP	0.87 (0.793-0.948)	0.801 (0.663-0.94)	0.738 (0.698-0.869)	0.72 (0.558-0.881)
R-R Hybrid	0.836 (0.75-0.922)	0.851 (0.726-0.976)	0.812 (0.725-0.9)	0.799 (0.655-0.943)
Xgboost				
NC	0.807 (0.725-0.89)	0.623 (0.428-0.818)	0.823 (0.746-0.901)	0.736 (0.572-0.9)
AP	0.834 (0.748-0.921)	0.768 (0.597-0.939)	0.839 (0.757-0.92)	0.72 (0.555-0.885)
PVP	0.786 (0.697-0.875)	0.742 (0.573-0.911)	0.82 (0.739-0.901)	0.816 (0.686-0.947)
BP	0.848 (0.764-0.924)	0.774 (0.637-0.912)	0.818 (0.738-0.8969)	0.691 (0.524-0.858)
R-R Hybrid	0.831 (0.747-0.915)	0.806 (0.666-0.947)	0.833 (0.75-0.916)	0.811 (0.648-0.975)

**Table:** 

(b)		
	Train cohort	Train cohort
Clinical logistic	0.726 (0.62-0.831)	0.705 (0.537-0.874)
C-R Hybrid logistic	0.785 (0.635-0.934)	0.81 (0.722-0.898)
Clinical Xgboost	0.808 (0.72-0.897)	0.71 (0.535-0.886)
C-R Hybrid Xgboost	0.833 (0.75-0.917)	0.803 (0.636-0.969)

NC, Non-contrast model; AP, Arterial phase model; PVP, Portal venous phase model; BP, Balanced phase model; R-R Hybrid, Combined radiomics model utilizing two distinct imaging sequences; Clinical, Model based solely on clinical indicators; C-R Hybrid, Clinical-radiomics hybrid model incorporating clinical variables (e.g., CEA, AFP, fibrinogen, tumor diameter) alongside radiomic features from 3D PVP and 2D BP images.

Logistic regression and XGBoost models were constructed using features extracted from all four sequences in both 2D and 3D imaging. The 2D combined model yielded an AUC of 0.851 (logistic regression, 95% CI: 0.726−0.976) in the test set, while the 3D combined model achieved an AUC of 0.811 (XGBoost, 95% CI: 0.648−0.975). These results indicated that both the 2D and 3D models demonstrated strong predictive performance for MVI. However, 3D radiomics did not provide a significant advantage and, in some cases, performed worse than the 2D model, particularly in the combined models.

Among the 44 different clinical features, LASSO regression identified four features (fibrinogen, CEA, AFP, and tumor diameter) for constructing a predictive model. The model based only on clinical features achieved an AUC of 0.71 in the test set (XGBoost, 95% CI: 0.535−0.886).

Finally, the 3D PVP and 2D BP models exhibiting the best performance were selected. These models were compressed into an imaging score, R-score, based on the λ coefficient. A predictive model was constructed that incorporated the R-score along with four clinical features identified. The clinical-radiomics combined model achieved an AUC of 0.818 in the test set (logistic regression, 95% CI: 0.654−0.981). Our findings suggested that while clinical features were able to predict MVI preoperatively, their predictive performance was inferior to that of radiomics. Furthermore, incorporating radiomics score and clinical features in models improved the predictive power. However, the combined model was not the most efficient.

Among the twelve groups of twenty-four models, eight logistic regression models outperformed others, while four XGBoost models demonstrated superior results. Overall, although XGBoost showed better performance in the training set, it did not exhibit an advantage over the logistic regression models in the validation.

**Figure 4:**
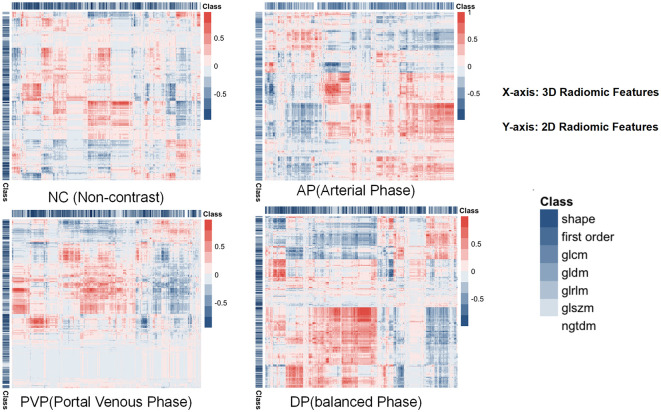
Illustrates the effects of each feature value on the model output and the importance of the features that contribute most to predicting MVI, with the total contribution scores of all features summing to 1. The combined 2D and 3D models are used to interpret the selected features for model construction. The upper part of the figure shows the 2D combined model and the lower part shows the 3D combined model. The SHAP values along the x-axis are color-coded, with high positive SHAP values (red) indicating a positive correlation with the prediction of MVI, and low negative SHAP values (blue) indicating a negative correlation. The point cloud plot displays the SHAP values of each feature for every individual sample. The horizontal axis represents the SHAP values, where positive values contribute to an increased prediction and negative values contribute to a decreased prediction, and the vertical axis lists the features ranked by importance. The color of each point reflects the magnitude of the feature value for the corresponding sample, with red indicating high feature values and blue suggesting low feature values. The numbers following the features represent the sequence from which they are derived: none indicates BP, 1 indicates PVP, 2 indicates AP, and 3 indicates unenhanced phase.

Subsequently, Shapley analysis was performed on both the 2D and 3D combined models. **Figure 4** presents the key radiomic features selected for constructing the models, along with their classification and contribution rankings. The results showed that the 2D combined model predominantly selected features from BP, whereas the 3D model favored features from the PVP. In contrast, among the single-sequence models, the performance for both 2D and 3D models was the best. Furthermore, there was significant feature overlap across different sequences, suggesting substantial collinearity among the selected radiomic features from various sequences. This indicated that the optimal features identified by LASSO largely originated from a single sequence. This finding contrasted with the results illustrated in the heatmap for the overall feature correlations.

In the 2D combined model, the contributions of various features were relatively balanced. However, in the 3D combined model, a single feature, wavelet-LLH-glszm-Small-Area-Emphasis, contributed over 60%. One of the dominant features in the model was derived from wavelet decomposition and GLSZM, which typically reflects the presence of numerous small and homogeneous grayscale zones at specific scales and orientations. This radiomic feature represents subtle intratumoral texture heterogeneity, which is closely associated with pathological characteristics such as necrosis, fibrosis, and MVI. These factors are generally indicative of tumor aggressiveness and poor prognosis. Therefore, the predominance of this texture feature in the radiomics model may be attributed to its capacity to capture the intricate spatial heterogeneity within the tumor, thereby enhancing the model’s ability to predict MVI accurately. However, over-reliance on a single feature can undermine the model’s robustness. If this feature is influenced by external conditions (e.g., image quality, data acquisition errors, or patient condition variations), the model’s performance may drastically decline. This could be one reason why the performance of the combined model incorporating 3D features did not meet expectations.

## Discussion

In the single-sequence radiomics models, the overall performance between 2D and 3D models was similar. A possible reason for this is the overlapping data characteristics between 2D and 3D ROIs. In liver cancer radiomics, some features across different slices of certain sequences may be quite similar. Although the 3D model processes all slices, it does not capture significantly different information. The imaging features of MVI may already be adequately reflected in the local regions of the tumor. Additionally, a large number of texture features and wavelet transform features were extracted, and SHAP analysis also indicated (**Figure 3**) that for some sequences, MVI prediction mainly relied on texture features rather than spatial features, thereby reducing the need for spatial information. As a result, the spatial information contribution of the 3D model may be weakened.

In the field of liver cancer, no related research has discussed the advantages and disadvantages of 2D and 3D ROIs. However, similar conclusions have been reached in comparable studies in other cancers. In lung cancer, Zhang et al. (18) reported that the predictive performance of 2D and 3D models using the CT plain scan phase did not differ across various tasks (such as lymphovascular invasion (LVI), pleural invasion (VI), and pT staging prediction) (19). Another study compared the performance of 2D and 3D MRI features in meningioma grading and found similar predictive performance between the two (20). A multi-center study compared the performance of 2D and 3D CT radiomics in gastric cancer characterization and concluded that 2D features were slightly superior in most tasks. These studies noted common disadvantages of 3D models. 3D image annotation is performed layer-by-layer, which is time-consuming, labor-intensive, and noisy. Manual delineation of 3D tumors often results in unclear boundaries, which can affect predictive accuracy. Additionally, the Z-axis thickness in 3D ROI delineation is typically coarser than the planar resolution along the X and Y axes, leading to loss of detailed information due to inconsistent spatial resolution, while 2D imaging can capture the clearest information in a single maximal cross-section.

However, the aforementioned reasons cannot explain why the 2D ROI model demonstrates better predictive performance than the 3D model during BP, while the 3D ROI model outperforms the 2D model during the PVP. A correlation analysis of both 2D and 3D features was conducted, as shown in **Figure 3**. The correlation between 2D and 3D features was strongest during the BP and weakest during the PVP, which aligned with the results of the predictive models. Based on this correlation analysis, it can be speculated that the observed findings may be attributed to the intrinsic characteristics of HCC. Compared to other tumors, HCC may be more heterogeneous. Its growth pattern is typically irregular and infiltrating, often extends beyond a single plane and tends to invade surrounding tissues (1). Additionally, HCC is often highly vascularized (21), especially during the arterial and PVPs, where the tumor shows significant enhancement in blood flow. These vascular characteristics are expressed in a 3D distribution across different layers. The PVP images better reflect the spatial structure, density variations, microvascular distribution, and perfusion differences inherent to HCC heterogeneity. The 3D ROI can provide more comprehensive information than a single 2D plane.

During the BP, the 2D model outperformed the 3D model. This may be due to the unique features of the BP in predicting MVI: the BP better reflects the tissue permeability characteristics associated with MVI (22), where the contrast agent achieves relative balance between the tumor and surrounding tissues. At this point, the tissue permeability of the tumor and microvascular characteristics become more prominent. In 2D images, these local texture and density variations can be more clearly presented, effectively offering features required for MVI prediction. Additionally, the potential “information dilution” issue of 3D integration may be another explanation. The 3D model combines features from multiple slices, which may dilute the predictive power of local features during the BP.

Regardless of the 2D or 3D model, wavelet transform features played a crucial role. This suggests that both models rely on local texture details from certain frequency sub-bands. The stronger results from the 2D model imply that these texture details may not exclusively come from the local regions where MVI occurs; texture details from other areas of the tumor can also reflect the presence of MVI. From a radiomic perspective, this provides further evidence for the heterogeneity of liver cancer.

In comparison, a model based on clinical features was developed using the same method. The model also demonstrated predictive ability for MVI preoperatively. Among 44 clinical indicators, tumor size, and AFP had the most significant predictive power, which is consistent with most prior studies (23, 24). However, the clinical feature-based model did not outperform the radiomics-based models from any sequence.

Next, the optimal radiomic features from the 2D BP and the 3D PVP in the single-sequence models were combined with clinical features to construct a multimodal radiomics-clinical combined model. Our results indicate that incorporating radiomic scores and clinical features in a model enhances the predictive ability. This conclusion aligns with findings from several previous studies. Xia et al. (25) constructed a clinical-radiomics semantic feature-radiomic score prediction model, which predicted MVI status with an AUC of 0.86 (95% CI: 0.79, 0.92), and the external test set yielded an AUC of 0.84 (95% CI: 0.78, 0.91). Compared to the pure radiomic model, the combined model provided a greater net benefit within a reasonable threshold probability range. A multicenter study by Xu et al. Xu (9) has suggested that the accuracy of the radiomic model in predicting MVI outperforms the clinical logistic regression model based on AST, tumor size, and AFP (AUCs in the validation cohort were 0.750 and 0.648, respectively). However, in this study, it was not the optimal predictive approach, which may be attributed to various factors, including sample size and the weaker noise resistance of the 3D model.

In this study, LASSO was employed to select 63 features from a total of eight sets of imaging data, with a significant number of wavelet transform features identified. Notably, some of these wavelet features appeared repeatedly across different sequences of 2D and 3D images. This suggests that the prediction of radiomics-based model for MVI is highly dependent on local texture features and that there exists a strong collinearity between certain features across different sequences of contrast-enhanced imaging. A correlation analysis of the features was conducted, and a heatmap was plotted to further substantiate this observation.

The findings by Ni et al. (26) indicate that the dimensionality reduction and model construction methods employed for radiomic features can impact the predictive performance of the resulting radiomics-based models. LASSO dimensionality reduction combined with gradient boosting decision trees (GBDT) yields the highest prediction accuracy, and the LASSO + GBDT method outperforms other approaches when the threshold probability exceeds 0.22. Both GBDT and XGBoost are ensemble methods that use multiple weak learners (typically decision trees) to enhance model performance. XGBoost is an extension of GBDT that focuses on improving performance and efficiency. It incorporates several enhancements over the GBDT algorithm, including regularization terms, more efficient node splitting, and learning rate decay, which result in superior speed and performance compared to traditional GBDT (27).

Based on Ni et al.’s conclusion, a LASSO + XGBoost predictive model was constructed, with a logistic regression model used for comparison. However, in this study, although XGBoost demonstrated better performance on the training set, it did not show an advantage over the logistic regression model in the validation set. This result may be attributed to the complexity of XGBoost’s tree model structure, which showed slight overfitting in the validation set. If the relationship between CT imaging features and liver cancer MVI is not highly nonlinear, the advantages of XGBoost may not be fully realized, potentially even leading to reduced predictive performance. Logistic regression might be more suitable for this relatively simple feature representation. Notably, in comparison with the logistic regression model, the XGBoost model often performed better with the 3D models, likely due to the higher complexity of the 3D datasets. On the other hand, XGBoost models involve numerous parameters (such as learning rate, tree depth, and regularization parameters), and achieving optimal performance requires careful parameter tuning. Since the investigators in this study were clinicians with limited experience in tuning complex machine learning models, this may have contributed to the suboptimal performance of the machine learning models.

Overall, the radiomics models based on various sequences of contrast-enhanced CT images demonstrated a certain degree of predictive ability. The radiomics combined model outperformed some single-sequence models, though it did not achieve the best results. Among single-sequence models, the 3D PVP model exhibited the highest accuracy, while the 2D BP model showed the highest AUC. Ma et al. (28) conducted a study involving 157 patients with histologically confirmed HCC, with or without MVI, and found that radiomics features from AP, PVP, and BP of enhanced CT imaging could all be used to construct predictive models for liver cancer MVI They found that the PVP offered superior predictive performance compared to AP and BP, as well as multi-sequence models. Our findings are partially consistent with the conclusions by Ma et al.; however, in their study, the combined model directly combined multi-sequence radiomic features, achieving high AUC in the training set, but with suboptimal accuracy, specificity, sensitivity, and AUC in the validation set. A preoperative radiomics model for MVI prediction (29) was similarly constructed, but the training and testing datasets showed considerable differences, which may have been caused by a mismatch in the number of features and the sample size of the training set, leading to model overfitting. Their conclusions are not sufficiently rigorous.

This study has several limitations. The single-center retrospective design inevitably leads to sample selection bias. Additionally, with a relatively small sample size, the advantages of more complex combined models are restricted due to the limited training data. Furthermore, our predictive model did not incorporate semantic features based on imaging interpretations by physicians. Moreover, variations in clinical issues, as well as image acquisition and reconstruction protocols, may influence the results. Therefore, further validation is required to improve the generalizability of the conclusions to other diseases and clinical contexts.

## Conclusion

This study successfully developed preoperative predictive models for MVI in HCC patients based on 2D and 3D radiomic features from contrast-enhanced CT imaging. The performance of different ROI delineation methods and imaging sequences was systematically compared. The results indicate that the 2D PVP model excels in capturing local texture features, while the 3D AP model demonstrates greater advantages in reflecting tumor spatial heterogeneity. Overall, the radiomic model outperforms traditional clinical-based predictive models, although the 3D model did not show a significantly better overall predictive performance than the 2D model.

The study further highlights the sensitivity of radiomic features to tumor heterogeneity, particularly in the expression of texture and spatial characteristics. Wavelet transform features are extensively selected and significantly influence prediction results. This underscores the pivotal role of radiomics in capturing the complexity and heterogeneity within tumors. This research provides robust evidence for radiomic studies in HCC, advancing preoperative MVI prediction techniques and offering critical insights for personalized and precise diagnosis and treatment of liver cancer.

## Data Availability

The original contributions presented in the study are included in the article/Supplementary Material. Further inquiries can be directed to the corresponding authors.
